# Animal roles and traces in the history of medicine, *c.*1880–1980

**DOI:** 10.1017/bjt.2017.3

**Published:** 2017-03-20

**Authors:** ANGELA CASSIDY, RACHEL MASON DENTINGER, KATHRYN SCHOEFERT, ABIGAIL WOODS

**Affiliations:** * Land, Environment, Economics and Policy Institute (LEEP), Department of Politics, University of Exeter, Lazenby House, Prince of Wales Road, Exeter, EX4 4PJ, UK. Email: a.cassidy@exeter.ac.uk.; ** Office of Undergraduate Studies, University of Utah, 195 Central Campus Dr, Salt Lake City, UT 84112, USA. Email: rachel.mason.dentinger@utah.edu.; *** Department of History, Kings College London, Strand Building, Strand, London WC2R 2LS, UK. Email: kathryn.schoefert@kcl.ac.uk.; **** Department of History, Kings College London, Strand Building, Strand, London WC2R 2LS, UK. Email: abigail.woods@kcl.ac.uk.

## Abstract

This paper argues for the need to create a more animal-centred history of medicine, in which animals are considered not simply as the backdrop for human history, but as medical subjects important in and of themselves. Drawing on the tools and approaches of animal and human–animal studies, it seeks to demonstrate, via four short historical vignettes, how investigations into the ways that animals shaped and were shaped by medicine enables us to reach new historical understandings of both animals and medicine, and of the relationships between them. This is achieved by turning away from the much-studied fields of experimental medicine and public health, to address four historically neglected contexts in which diseased animals played important roles: zoology/pathology, parasitology/epidemiology, ethology/psychiatry, and wildlife/veterinary medicine. Focusing, in turn, on species that rarely feature in the history of medicine – big cats, tapeworms, marsupials and mustelids – which were studied, respectively, within the zoo, the psychiatric hospital, human–animal communities and the countryside, we reconstruct the histories of these animals using the traces that they left on the medical-historical record.

## Introduction

One of the most striking recent trends in the historiography of modern medicine is the attention paid to animals and their diseases. This has resulted in historical biographies of diseases such as anthrax, rabies, tuberculosis, salmonella, trypanosomiasis and bovine spongiform encephalopathy, which identify animals as key transmitters of infectious diseases to humans, and subjects of control measures aimed at promoting human health.^1^ It has brought animals into histories of medical science, which demonstrate their experimental fashioning into surrogate humans or ‘models’ that helped to elucidate bodily structure, function and the nature and microbial causes of disease.^2^ Authors also reveal the roles played by animals as sources and standardizers of biological material for use in humans, and as testing grounds for human surgical interventions.^3^ The considerable resistance that these experimental practices inspired is well documented,^4^ as is the manner in which medical scientists fashioned animals into ‘the right tools for the job’ by reshaping their bodies and environments.^5^

As part of a wider ‘animal turn’ in history,^6^ this scholarship has illuminated the more-than-human foundations of medical knowledge, and the extent to which human health depended upon that of animals.^7^ It has thereby bolstered legitimacy for animals as specifically medical (rather than zoological or veterinary) historical subjects. However, while they increasingly pay attention to animals, the accounts produced by medical historians remain largely human-centred. Animals usually feature as passive objects acted on by humans,^8^ whose significance to medical history lay in the ways that they enhanced or challenged the health of humans by acting as experimental material or by transmitting diseases.^9^ While animals certainly played these roles, this article argues that this is not all they did, and that in order to advance historical understandings it is necessary to think more broadly about their contributions to medicine, and to engage more deeply with their lived experiences and capacity to act as agents of historical change.

There are various explanations for existing medical-historical approaches to animals: the traditional anthropocentricity of the discipline of history, the popularity of public health and experimental medicine as subjects of medical-historical analysis, and the fact that in these fields – as in medicine today – animals did play important, visible roles as disease transmitters and experimental material. Prevailing perceptions of what constitutes medical history are also responsible. It is as if the claim made by the late Roy Porter in 1993 still stands: ‘in the academic world, it is automatically assumed that a “historian of medicine” is a person who works on the history of human medicine’.^10^ By extension, the study of sick animals is assumed to be part of veterinary history, while animals in general are located within the history of biology. This compartmentalization has resulted in a narrowly anthropocentric framing of ‘medicine’ that separates it artificially from other domains concerned with the history of animal health.^11^

Tools and concepts for creating a more animal-centred history of medicine have been developed within the burgeoning fields of animal and human–animal studies. These draw on elaborations of actor-network theory to investigate how animal bodies, minds and behaviours shaped the perceptions and actions of the people who studied them, and use the findings to reframe established human-centred narratives.^12^ There is mounting archival evidence that could be used for this purpose. Uncovered by the authors in the course of a five-year research project on the relationships between human and animal health,^13^ it locates animals historically within fields that straddled the boundaries between human medicine, veterinary medicine and the life sciences. Within natural history, parasitology, morbid anatomy, comparative medicine, ecology, ethology and food production, researchers studied animals not simply because of their capacity to shape human health, but also in the hope of illuminating the general nature of health and disease, or advancing animal health as an end in itself. Within these investigations, animals were approached not simply as disease transmitters or experimental material. They were also awarded many other, historically overlooked roles such as spontaneous (rather than laboratory-created) analogues of human diseases, tools for thinking comparatively about medical and biological problems, shapers and products of disease environments, disease victims, forgers of research networks, and carriers of personal and professional ambitions.

We contend that bringing the approaches of animal and human–animal studies to bear on this type of archival evidence will enable historians, first, to document the historical experiences of animals by analysing them as medical subjects important in and of themselves; second, to develop new insights into how animals shaped (and were shaped by) medicine; and third, by following animals beyond public health and experimental medicine into the borderlands of human medicine, veterinary medicine and the life sciences, to generate new perspectives on what constituted medicine at particular points in time.

The remainder of this article will illustrate and elaborate these claims through the presentation of four short historical vignettes. Each focusses on a distinctive, historically under-studied scientific context in which diseased animals were constructed as medical scientific subjects through the application of diverse investigatory methods: zoology/pathology, parasitology/epidemiology, ethology/psychiatry and wildlife/veterinary medicine. Drawn from the authors' individual research programmes, which span the study of animals in health and medicine, in various countries over the 1880–1980 period, the vignettes highlight four attendant spaces for the study of animals: the zoo, the psychiatric hospital, human–animal communities and the countryside, and focus, in turn, on the histories of monkeys/big cats, tapeworms, marsupials and mustelids.

These vignettes are not intended to offer a complete history of animals in medicine or to document either progression over time or variation by place. Collectively, their purpose is to offer distinctive examples of disease investigations involving contexts, spaces and species that are almost entirely neglected by existing medical-historical literature. Through highlighting the diverse roles that animals played in these investigations, and the implications for animals as well as for humans, we hope to inspire readers to think more creatively and less anthropocentrically about the history of animals and medicine, and to participate in this new research agenda.

Adopting an animal-centred approach to the history of medicine requires us to engage with the question of how to construct accounts of non-verbal beings when the only records that survive of them were created by humans. This issue has stimulated much reflection and comment within animal and human–animal studies.^14^ For some commentators, surviving historical records are merely cultural representations that can reveal little about animals' authentic lives.^15^ In the context of medical history, however, they can clearly be more than that, for life and disease left biological marks on animal bodies, which human investigators examined, manipulated, interpreted and recorded. For Etienne Benson, such records constitute ‘animal traces’, and are key to developing ‘a richer history – a true “animal history”’.^16^

Using Benson's concept as a starting point, this article constructs the history of animals within medicine through a multi-layered analysis of the traces they left on the historical record: from the immediate material remains of diseased animal bodies, to the narratives, statistics and images that human investigators created from them, and the knowledges and practices that derived from analyses of these creations. Like historians of the body, we understand the events, processes and concepts associated with animal traces to be simultaneously corporeal, imagined and social,^17^ thus avoiding unhelpful dichotomies between ‘real’ animal histories and ‘constructed’ human representations of animals. This approach allows us to follow animals on their journeys through the history of medicine, to unpack the methods used to investigate them, and thereby to offer important insights into their lived experiences and the ways in which they contributed to the development of medical ideas, practices, policies, institutions and careers.

## Rickety monkeys and deformed lions

Tucked away in a corner display of the Royal College of Surgeons Hunterian Museum in London are the limb bones of a monkey. The shafts of the long bones are curved and their ends are thickened and malformed.^18^ Within the museum store sits another, entire monkey skeleton, exhibiting fractures to its limbs, a distorted sternum and pelvis, and a thickened vertebral column. The accompanying case report suggests that the monkey suffered greatly. Fully grown and kept in captivity, its condition deteriorated gradually. The lower limbs became paralysed, there was urinary and faecal incontinence, and the abdomen became distorted.^19^ The diagnosis in both cases was the same: rickets.

These bony traces of rickety monkeys were separated from their fleshly bodies and deposited on the historical record by John Bland Sutton, future baronet and president of the Royal College of Surgeons (1923–1925). He obtained them in 1883 from monkeys housed within the London Zoological Gardens.^20^ Monkeys were not the only rickety animals he encountered within the zoo. Traces of many other species feature in the written reports of meetings where he displayed their bodies, in the specimens he deposited within museums, in the copious articles he contributed to the medical and zoological press, and in the discussions his findings provoked within the medical profession. These traces illuminate not only the lives and afterlives of certain inhabitants of Britain's most famous zoo, but also the activities and concerns of the men who studied them, and turned their fates to human advantage.^21^

As the impoverished son of a taxidermist, Bland Sutton had paid his way through medical school by working as a demonstrator and private teacher in anatomy.^22^ When newly qualified, he began to perform post-mortems on all the animals that died in the London Zoological Gardens. Although not the first doctor to conduct such examinations, he was far more systematic than his predecessors, incorporating them into a personal research programme that involved the dissection of 12,000 human and animal subjects between 1878 and 1886. For Bland Sutton, the dead animals of London Zoo served as vehicles of professional advancement. His examination of their bodies enabled him to mingle socially with prestigious members of the London Zoological Society. His insights into animal diseases resulted in invitations to address London's many medical societies, whose reports – when published in the medical press – significantly enhanced his professional profile.^23^ Animals ceased to perform this role only after he won the post of assistant surgeon to the Middlesex Hospital in 1886, and began to focus more intensively upon human patients.^24^

Bland Sutton first entered the zoo at the behest of the Pathological Society of London (PSL). Numbering over six hundred members – mostly private practitioners and London hospital doctors – the PSL was an organization dedicated to ‘the cultivation and promotion of pathology by the exhibition and description of specimens, drawings, microscopic preparations, casts or models of morbid parts’.^25^ While diseases of animals had long featured in its meetings and journals, interest in their relation to human diseases rose up the agenda in the later 1870s. This was due to a series of enthusiastic presidents, as well as wider bacteriological and epidemiological investigations encouraged by new germ theories, which illuminated the animal-to-human transmission of certain infectious diseases.^26^ Rather than embracing the new experimental pathology that employed animals within laboratory settings to investigate infectious disease,^27^ the PSL elected instead to apply older, morbid anatomical methods to all forms of disease. As a scientific institution, where the circumstances of disease and death were closely monitored, and which boasted ample facilities for conducting post-mortems on the many animals that died, the zoo seemed an ideal site of enquiry.^28^

Under the PSL's direction, Bland Sutton approached diseased and dead zoo animals as points of comparison with humans. In contrast with laboratory-based medical scientists, who deliberately created diseased animals that could substitute for humans in experiments, he focused on animals that suffered spontaneously from disease, and sought, by comparison, to determine the similarities and differences between their diseases and those of humans. Whereas experimentalists relied on small rodents and dogs, Bland Sutton was attracted particularly by monkeys, owing to their proximity to humans on the zoological scale. Like experimentalists, he referred to his work as ‘comparative pathology’, but unlike them, his work has passed unnoticed by historians.^29^

In the fourteen months from December 1881, Bland Sutton examined the bodies of a hundred dead monkeys. He discovered that, contrary to popular belief, most had died of bronchitis, not of tuberculosis (which, as in humans, was regarded as the commonest cause of death at the time). Unexpectedly, he found that the second most frequent cause of death was rickets. This, too, was a major health concern in humans. Found especially in poor, urban children, it was characterized by softening and deformity of the bones, stunted growth, a large head, a misshapen chest, twisted long bones and enlarged wrists and ankles. The cause was uncertain, although observers suggested various contributing factors, such as faulty diet, poor hygiene, inheritance, lack of exercise and lack of fresh air and sunlight. The existence of rickets in zoo animals seemed to offer a perfect point of comparison, a ‘side light of no mean power’ to elucidate the disease's manifestations in humans.^30^

To this end, Bland Sutton retained and studied all of the bodies of monkeys that died of rickets in the zoo during 1882. He learned to distinguish different forms of rickets occurring at different ages, and to identify analogous conditions in humans. Moving beyond the comparison of pathological changes, he began to report upon the symptoms that diseased monkeys experienced in life, such as diminished activity and paralysis of the lower limbs. Monkeys responded by using their arms as crutches until these began to bow under the weight. They eventually became paraplegic, and suffered incontinence and priapism. After three to four months, death intervened, usually from bronchitis.^31^

Bland Sutton's comparative project grew larger still as he began to identify other animal species that suffered from the disease. In an 1884 paper to the *Journal of Anatomy and Physiology* which he illustrated with line drawings of affected bones from monkeys, a baboon and a sloth bear, he reported its presence in half of the zoo's dead carnivores, as well as in many rodents, birds and lizards.^32^ Remarking that rickets was ‘as common, or even more frequent, among wild animals in captivity than among children’,^33^ he began to draw epidemiological analogies between the conditions of animal life within the zoo and those experienced by human sufferers. He identified restricted exercise, exposure to climate and unsuitable diet as common contributing causes, together with the failure of mothers to suckle their human and animal offspring.^34^

Bland Sutton also extended his enquiry to the clinical management and prevention of rickets. Here, he awarded lions the role of patients, and sought to improve and preserve their health, ultimately for the benefit of the London Zoological Society. Lions were costly animals and popular with fee-paying visitors on whom the society depended for the zoo's financial survival. Consequently, their health was a matter of considerable concern. Their offspring had proved virtually impossible to rear within the confines of London zoo. Many were born with cleft palates and did not survive for long. Others developed signs of rickets after keepers removed them from their mothers for fear of harm. Bland Sutton noted that both adults and cubs were typically fed on old horse carcasses, whose bones were generally too tough for lions' teeth. If pregnant lions were fed goat-flesh and soft bones, cleft palates in the offspring did not occur. Moreover, rickety cubs quickly recovered when pounded bones and cod liver oil were added to their diets.^35^

Bland Sutton did not publish a formal account of these findings, perhaps because, from the zoo's perspective, cod liver oil supplements cost as much as replacing a lion.^36^ Dietary changes were not adopted and the disease continued to occur in lions, as shown by the ongoing deposition of their rickety skeletons in the RCS Hunterian Museum.^37^ However, some medical men became very excited by what Bland Sutton's work implied about the causes and management of rickets in humans. Speaking at the Diseases of Children section of the British Medical Association's 1888 Annual Meeting, Dr Cheadle, a senior physician to the Hospital for Sick Children, Great Ormond Street, declared Bland Sutton's dietary experiment ‘a crucial one, and … conclusive as to the chief points in the aetiology of rickets’.^38^ It showed that rickets occurred when diets were deficient in fat and bone salts. This became the accepted view of the disease. Transcending the zoological, anatomical and pathological communities addressed by Bland Sutton, traces of rickety lions began to feature in discussions of human rickets and infant-feeding practices. Their fates also provided the jumping-off point for Edward Mellanby's subsequent discovery that the key anti-rachitic component was a substance found particularly in animal fat, later named fat-soluble vitamin D.^39^ In this way, spontaneously diseased zoo animals became unwitting participants in the advancement of human health, a role analogous to – but distinctively different from – that concurrently performed by the intentionally diseased animal subjects of a quite different, experimental form of ‘comparative pathology’.

## Restless marsupials

A North American marsupial, an opossum (*Didelphis marsupialis*), was stalking an institute of brain anatomy in 1960. Her frantic roaming animated the institute's laboratory at the Waldau, the largest state-run psychiatric hospital in the Swiss canton of Bern. Her pacing left traces in a short paper written by the Waldau's neuropathologist Giorgio Pilleri (born 1925). It reminded him of stereotypical behaviour seen in long-term hospitalized patients, and the ‘quasi-neurotic’ behaviour patterns of mammals held in captivity which German-speaking psychiatrists and zoologists had discussed at length in the 1930s.^40^ Pilleri described how the opossum had quieted once she was no longer kept awake during the day contrary to opossums’ nocturnal habits. His account of her movements was a rare, inadvertent portrait of the laboratory's material conditions, otherwise black-boxed in his academic papers. Moreover, in her wandering, the opossum had unintentionally exposed the mid-twentieth-century preoccupation with the effects of long-term hospitalization, behaviourist approaches to mental health and its treatments, and socio-environmental causes of psychiatric disorders.^41^

The opossum shared the laboratory with a South American paca (*Cuniculus paca*) who came when called and moved through the building freely, climbing the stairs to the first floor, leaving droppings and marking territory. Within weeks the animal had appropriated part of Pilleri's office and was defending a hallway bookcase by growling.^42^ When deprived of her nesting site, the paca ‘lost drive, was listless, seemed somehow different than usual’, Pilleri remarked in a second paper.^43^ Paca and opossum were pets and curiosities, which Pilleri's observations framed as spontaneous analogues to human behaviour. He collected, described and classified observations of living animals as well as of post-mortem specimens, an approach that echoed long-established practices in natural histories.^44^ The animals’ provenance reinforced such links. The opossum had been captured in a Mississippi wildlife refuge while still in her mother's pouch, the paca acquired with the help of a Dutch zoo.^45^ Pilleri scrutinized the behaviour of other rodents, including aplodontia (North American mountain beaver) during field trips.^46^ However brief, the studies were valid according to Pilleri, because they disclosed habits of rare, hitherto under-studied, animals. By embracing species diversity, he sought to reveal the range of healthy behaviour within a taxonomic order. The paca thus was to contribute to understanding the spectrum of rodent behaviour beyond mice and rats which populated behaviourist laboratories and whose behaviour – and brains – were far more familiar to researchers.

While Pilleri acknowledged that his institute was in many ways deeply unsuitable for ethological work, he suggested that his laboratory's ‘utterly unnatural … conditions’ were also a potential asset.^47^ They demonstrated the innateness of the paca's marking of territory or the opossum's nocturnal habits. Yet conditions were poor and the laboratory was crowded. Its neuropathological collection threatened to engulf all spaces and surfaces, and the smells from the institute's unrefrigerated autopsy suite were overwhelming.^48^ Cantonal financial resources, however, were limited. The institute's director, Ernst Grünthal (1874–1972), therefore appealed for external funding, contacting his collaborators at the Swiss pharmaceutical company JR Geigy AG, in Basel. Grünthal had Pilleri's opossums and beavers in mind when he wrote that systematically observing animals might uncover ‘more advanced[,] spontaneously occurring[,] psychic animal functions’ and therefore previously unknown means to assess drugs.^49^ When the institute's new building opened five years later, substantially financed by Geigy, it included in- and outdoor spaces to observe birds and rodents as well as clinical spaces to observe human subjects volunteering in pharmaceutical trials.^50^ Tentative and brief in the record, the opossum's traces therefore also substantiated researchers’ career paths and professional identities.

Did the laboratory's furry inhabitants correspond to a more general alignment of psychiatry and animals around 1960? Psychiatric hospitals had long been criss-crossed by animals. They had been considered food and economic resources, feared as disease carriers and public-health nuisances, tolerated as companions; and it had been common to see those afflicted by mental illness as ruled by animal passions. As the opossum paced, Geigy was establishing new research protocols that relied on animal experiments to assay psycho- and neuroactive drugs.^51^ Yet the traces of the opossum and paca also demonstrated another, non-experimental research direction. Post-mortem the disconcerted opossum might have provided the brain specimen for one of Pilleri's papers in comparative brain anatomy which included photographs of opossum brains and brain tissue.^52^ The opossum was known to have a primitive brain, but it had opposable digits and motor skills usually associated with more developed brains.^53^ Did species-specific behaviour correspond to brain or nerve cell composition? Pilleri refuted the link, concluding elsewhere that manual dexterity was indicative of neither an animal's level of brain development nor its ability to shape the environment.^54^ In later papers, he stated that brain size did not determine cultural sophistication.^55^ Overly specialized brains could indeed be detrimental in evolutionary terms, because they limited avenues of adapting to, and mastering, the environment.^56^

The Bernese opossum therefore contributed to a study of instincts that was inspired by the German biologist Konrad Lorenz's mid-twentieth-century ethology, neurologists’ cerebral localization studies, and Darwin's *The Expression of the Emotions in Man and Animals* (1872). Konrad Lorenz had postulated that instinctual behaviour was innate and fixed in form, and therefore amenable to trans-species comparison. Pilleri argued that this ethological approach systematized clinical observations by grouping symptoms in ‘natural’ orders.^57^ Pilleri built on earlier neurological theories that nervous system and behaviour retained archaic roots even as more advanced functions matured. Phylogenetically younger brain components inhibited older structures, but illness or dysfunction could unpick these links.^58^ Pilleri's neurological papers thus related automatism seen in patients with severe dementia to infants’ suckling; one patient's ‘climbing motions’ were a return to clinging to a mother's fur.^59^ Photographs of patients and primates mid-movement illustrated these connections. Pilleri's papers, moreover, showed that this behaviour, uncalled-for in adult humans but not in infant primates, mapped onto brain lesions identified post-mortem. Scrutinizing animal and human behaviour was therefore diagnostically useful. To closely observe animals was to closely observe humans – and vice versa.

However unusual the marsupial was in the psychiatric hospital, Pilleri was not alone in linking ethology, psychiatry and neurology.^60^ A chapter in a 1964 German handbook of psychiatry thus foresaw a brain-based ethology providing psychiatry and psychoanalysis with a much-needed biological foundation.^61^ Even though ethologists were unable to capture the ‘specifically human’ aspects of human behaviour or its disorder, they could underpin a biological psychiatry equally attuned to soma and psyche.^62^ Pilleri's opossum paper also cited interwar authors who had argued that humans and animals alike could be ambivalent, latently neurotic and torn by wants. Some envisaged animal experiments to examine the emergence of neuroses in varying environmental conditions, a research direction subsequently taken up in post-war enquiries.^63^ Pilleri himself pursued few experimental studies in the early 1960s, conceiving of paca and opossum as spontaneous analogues, tools to re-catalogue human behaviour and illness.

The ‘neurotic-like’ opossum and the growling paca inhabited a psychiatry wrestling with demands for patient autonomy, new attempts to localize mental illness in the brain, changing treatment models, and the hope to have finally become a therapeutic discipline. Opossum and paca, moreover, occupied the laboratory at the same time as veterinarians’ attention was turning to animal mental health, therapy animals and symbiotic relationships between companion animals and owners.^64^ The animals confronted those who saw psychiatry as exploitative and oppressive. Were psychiatric diagnoses not socially constructed? fumed R.D. Laing, Thomas Szasz and others. For them, ethologists’ projects to found psychiatric knowledge on non-human animals further dehumanized patients and removed their agency. Tracing the opossum and paca was therefore spurious, indeed dangerous. The frantic opossum was thus troubled and troubling as it wandered the hospital.

The opossum's movements *intra vitam* and post-mortem disclose the growing enmeshment of behaviour, brain and environment – and its challenges – in the second half of the twentieth century. Animals’ habits became framed as a resource to understand, and systematize, human behaviour, a project later extended by sociobiology and evolutionary psychology.^65^ Drawing animal and human health together co-constructed new views of mental behaviour and appropriate treatments. First applied to humans, these have been considered for animal health in the last decade. Antidepressants, once appraised with the help of laboratory mice, are being prescribed to companion, farm and zoo animals, and books discussing animal madness are popular.^66^ Following the opossum under observation in the psychiatric hospital traverses the assumed disciplinary boundaries of post-war psychiatry and its principles. However, odd, she hints at a wider, densely knit history of psychiatry, its cares and cures which goes beyond patient–doctor dichotomies.

## Parasitic tapeworms

In August 1965, surgeons in California carried out an operation on a five-year old boy, known to us only as ‘J.O.’ He had presented with acute abdominal pain that began when he leapt to the ground from the back of a flat-bed truck. Appendicitis was suspected. On opening the abdomen, doctors found that his liver and lungs were strewn with hydatid cysts, ranging in diameter from two to ten centimetres. His leap had ruptured a cyst in his liver and released the contents inside his body.^67^ These cysts were the products of a parasitic infection, undoubtedly transmitted to him by one of the many dogs that populated his family's ranch in the San Joaquin valley. Like many sheep ranchers of Basque descent, J.O.’s father, Salvador, differentiated amongst his dogs: while the majority were considered ‘workers’, and kept chained outside at night and treated more firmly, most ranchers also had at least one ‘favourite’, allowed more privileges and closer contact with their masters’ families.^68^ A single affectionate lick of the boy's face would have been sufficient to transmit the eggs of the tapeworm, *Echinococcus granulosus*. Hatching into larvae within J.O.’s intestine, they penetrated the intestinal wall and migrated throughout his body, settling in his lungs and liver, where they grew into cysts, which generated more larvae that spread and anchored to produce cysts in other organs.

The traces that *E. granulosus* left on J.O.’s body subsequently came to the attention of veterinarian Calvin Schwabe, professor of veterinary epidemiology at the University of California, Davis. For Schwabe, *E. granulosus* had been a vehicle for professional advancement for at least a decade. Trained also in parasitology and public health, he had built his scientific reputation on investigating the parasite while working as professor of parasitology at the American University in Beirut (AUB) and consultant to the World Health Organization.^69^ While rampant in the environs of Beirut, *E. granulosus* was practically unknown in California when Schwabe arrived there in 1966. Isolated hospital cases like J.O.’s had yet to be situated within a larger epidemiological picture, and parasitologists believed that most American infections had been contracted abroad.^70^ In 1967, an unexpected discovery challenged this presumption. While collecting cysts from sheep carcasses at a local abattoir, one of Schwabe's students identified what he believed to be *E. granulosus* cysts. The news reinvigorated Schwabe's research programme.^71^ In the Central Valley of California, geographically and culturally removed from Beirut, Schwabe would again observe a network of social and biological links between humans and animals. At the centre of these linkages, articulating existing associations and enabling new ones, was one diminutive but essential animal: the tapeworm *E. granulosus*. Moving from one animal body to another, it created corporeal connections between bodies – from which many other subsequent connections, both literal and figurative, were generated.

The traces that this diminutive animal left on the historical record enable the historian to reconstruct its social and scientific networks, the roles it played within them, and their implications for scientific understandings of parasites as well as for Schwabe's career. It may seem unusual to award a parasite the status of ‘animal’, yet for a biologist it is self-evident. While the nature of *Echinococcus* cysts was long ambiguous, tapeworms in general have been recognized as animals since the time of Linnaeus.^72^ Their zoological status was further solidified in the late nineteenth century when the study of parasitic helminths and protozoa split off from the study of bacteria – the former considered ‘animal parasites’, the latter ‘plant parasites’. As a member of kingdom Animalia, *E. granulosus* is known to share characteristics with many free-living animals, including a nervous system and sensory organs. Its key difference lies in its physiological dependence upon the host. On account of this dependence, it has been unusual – in non-scientific discourse – to award parasites the same degree of autonomy and agency as larger, more familiar animals. However, this has not been parasitologists’ perspective. Working at the intersection of medicine, zoology and natural history, they viewed *E. granulosus* and other parasites as animals in their own right, simultaneously performing the roles of disease threat, predator and symbiont.^73^ *E. granulosus* functioned also as product and shaper of its environment, an animal that created ecological connections between other animals, and moved opportunistically through the social connections created by humans.

At AUB, Schwabe had used the classic tools and approaches of parasitology to fashion *E. granulosus* into an object of laboratory experimentation, natural-history study, and public-health disease prevention.^74^ He thus sought evidence for the commonly held view that younger humans and animals were more susceptible to infection – although, typically, infections did not become apparent or symptomatic until years later, when cysts that the parasite generated became intolerably large or burst, leading to terrible pain or anaphylactic shock.^75^ Schwabe also investigated the physiological mechanisms in the host that allowed the parasite to thrive. As in much laboratory-based infectious-disease research, the researchers’ goals aligned with those of the parasite. Experiments aimed to ensure the parasite's survival and therefore enable it to proliferate and spread between hosts in the lab. While Schwabe's experiments intended to develop defences against the tapeworm and limit infections, he had first to nurture the parasite within his laboratory.

To a parasitologist, different host bodies constitute unique ecological environments for their primary research subjects, the parasites themselves; thus Schwabe sought to understand the tapeworm's ecological requirements and use this knowledge to create conditions in which *E. granulosus* would flourish.^76^ In so doing, he awarded his parasitic animals a form of agency similar to that which featured in the human domestication of animals. *E. granulosus* succeeded in the laboratory by engaging the cooperation and resources of researchers, and altering their practices and conceptions – a sort of ‘coevolutionary process’ whereby humans moulded animals, and animals moulded humans.^77^

After collecting cysts from a human brain, Schwabe gave them new life by injecting them into a laboratory population of mice, thus introducing them into a new ecological environment. The animal traces here may be read in the photographic evidence of the parasite's proliferation. A mouse's abdomen was cut open to reveal enormous clusters of cysts, while micrographs of cyst cross-sections showed the parasitic larvae multiplying on its interior.^78^ These traces made visible the extent to which the parasite altered the interior cellular landscape of the host. By making these signs of infection visible, Schwabe also made visible the agency of *E. granulosus*, evident not only in the way the parasite reshaped its environment, but also in the way it successfully concealed itself from the host animal for long periods so that the cells at the interface of parasite and host became difficult to differentiate.^79^

During this period, Schwabe collaborated with colleagues in the AUB chemistry department, measuring the movement of fluid and oxygen between the cyst and the host, to better understand how the parasite adapted to its environment.^80^ His published record of the parasite's respiration and how it actively controlled the fluid environment of the cyst brought its fundamental animal nature more clearly into focus. Belying its otherwise passive and inert appearance, the *E. granulosus* cyst was physiologically responsive to changes in its environment, and capable of altering that environment to suit its requirements.

Yet he was also considering the broader network of biological and social environments, contacts and exchanges that created opportunities for *E. granulosus* to move between hosts. The insidious nature of the clinical disease caused by *E. granulosus* made gauging its incidence of infection in the human population a challenge. To make the parasite more visible, he and his colleagues sought evidence of its traces in the surgical records of fifty-four Lebanese hospitals, tabulating the number of hydatid cysts found between 1949 and 1959. Strikingly, they found a high proportion of Christian patients – numbering twice as many as Muslim patients. The study, published in 1961, claimed that ‘religion was a real epidemiological factor’, and posited Muslim cultural prohibitions against close contact with dogs as a possible explanation. Later efforts to gather data further suggested that Christian families were more likely to keep dogs as family pets and encourage children to develop affectionate bonds with them.^81^ In invoking the importance of these familial, social norms and relationships alongside surgical data, Schwabe turned ephemeral connections between humans and canines into yet another type of animal trace: case studies and personal recollections, which recorded the parasitic animal's movement from one interior host ecosystem to another.

Schwabe's intense awareness of the importance of social relationships and cultural practices proved just as important in California in 1966. Following his student's discovery of hydatid cysts in sheep, he began to seek the parasite actively, discovering ubiquitous traces on hospital surgical records.^82^ These led him to the Central Valley sheep ranches and the communities into which ‘J.O.’ was born, which he identified as the main sufferers of echinococcosis in California, thanks to the transhumant sheep-herding practices that Basque ranchers had brought to the United States.^83^ Just as in Beirut, *E. granulosus* traversed a unique network influenced by the local peculiarities of human sociocultural practice. In California this network connected families and ranch hands to their sheepdogs and sheep, as well as to the deer and coyotes that roamed far beyond the domestic sphere.^84^ As both product and shaper of its environment, the tapeworm created bodily connections between other animals that already shared an ecological and social space, and simultaneously built relationships between them, which would leave bodily traces upon the victims, and literal traces in the work of their investigator, Calvin Schwabe.

## Tuberculous badgers

In April 1971, a farmer in the Gloucestershire Cotswolds brought the dead body of a wild badger he had found on his farm into his local government animal health office. Roger Muirhead, one of the veterinary officers there, conducted a post-mortem examination of the animal. He reported pathological lesions caused by tuberculosis, and identified its causal bacterium, *Mycobacterium bovis*, in fluids taken from the badger's lymph gland. The diagnosis was subsequently confirmed by scientists at the government's Central Veterinary Laboratory, and was immediately communicated to other officials and scientific experts within the Ministry of Agriculture, Fisheries and Food (MAFF).^85^

Within four years this badger had stimulated a major programme of laboratory, clinical, experimental and field investigations into ‘TB in cattle and badgers’ conducted by the ministry. It had also precipitated a series of laws on the protection and management of wild badgers, which in adapted form still remain in force. Given that in 1971 relatively little was known about the pathology, microbiology and epidemiology of bovine tuberculosis (bTB) in wildlife, or about the ecology and behaviour of badgers, how did one sick animal end up performing such significant roles in such a short space of time? What can the history of this animal and its tuberculous compatriots tell us about human–animal relations, environmental politics and animal health policy in the early 1970s, and what light can its traces shed on the repeating cycles of public controversy over bTB which have unfolded between scientists, veterinarians, conservationists, farming interests and politicians over the last five decades?

By the 1970s, MAFF had been attempting to control bTB in cattle for several decades, initially for the purposes of public health – because the meat and milk of affected cattle were a major source of tuberculosis in humans – and latterly to boost cattle health and productivity. The chronic nature of bTB meant that affected cows showed few overt symptoms until the disease was well advanced, by which time they were significant spreaders of infection. In the 1950s, the government had rolled out compulsory testing of cattle herds at regular intervals, with enforced culling of those affected.^86^ Together with the compulsory pasteurization of milk, this spelt an end to bTB as a major public-health threat. By the 1960s, bTB rates in cows had fallen substantially. MAFF declared a number of regions in the UK to be free of the disease, and its eradication was confidently predicted.^87^

However, pockets of infection persisted, particularly in Gloucestershire and Cornwall, where some farms experienced repeated outbreaks. Unable to explain this, MAFF dispatched a veterinary team to the West Penwith area of Cornwall to conduct a full epidemiological investigation of the area.^88^ This investigation was inconclusive, but shortly afterwards the fate of the Gloucestershire badger came to light. It seemed to prove what some vets and farmers had long suspected: that badgers could spread tuberculosis to cattle. Further evidence emerged the following year, when Muirhead and his colleagues examined fifty-five badger carcasses from the surrounding area and found eleven suffering from bTB.^89^ News about these unpredictably infected animals almost immediately generated public calls for badger extermination.^90^ Ministers of agriculture appeared sympathetic to these calls. The parliamentary secretary commented, ‘Fond as I am of badgers, I am quite clear that we could not permit a situation to continue in which they were proved carriers of TB’.^91^

However, other parties were less certain what the discovery of diseased badgers meant and what action should be taken. Members of the Nature Conservancy Council (NCC), the government body responsible for scientific advice on conservation issues, were concerned about a potential ‘widespread purge of badgers’.^92^ Discussions of badger extermination had been ongoing since the late nineteenth century and generated conflicts around the role of the badger: was it sport animal, vermin or victim of human cruelty?^93^ Public campaigns for further badger protection had been gathering pace since the mid-1960s, and several attempts had already been made to pass private member's bills to effect this.^94^ Given the localized nature of the bTB problem and the small number of animals involved, NCC officers were also sceptical of the badger's posited role as disease transmitter, claiming, ‘The evidence for the badger as a source of infection is therefore somewhat flyblown. If anything, badgers are more likely to have been infected from the cattle in the first place’.^95^ Unlike government veterinarians, who employed epidemiological methods of tracing disease outbreaks through geographical association,^96^ the zoologists and ecologists of the NCC saw only the coincidental colocation of sick badgers and cows, which proved nothing about the ultimate source of infection.^97^

The evidence was also treated as tentative by MAFF's in-house experts on wildlife, albeit for more pragmatic reasons. Its Pest Control Division (PCD) and associated Pest Investigation Control Laboratory (PICL) were primarily concerned with the badger's potential role as a pest animal that damaged human crops and food supplies. Since at least 1946 PCD had received regular correspondence implicating badgers in digging up gardens, spoiling and raiding crops, destabilizing riverbanks and stealing poultry. Field officers and scientists had followed up these complaints and generally concluded that culprits were most likely other wildlife species, or sometimes aberrant old or sick individuals described as ‘old rogue badger[s]’.^98^ Yet the occasional need to remove badgers meant that scientists at the PICL had investigated ways of controlling them. As badgers were not a legally designated ‘vermin’ species, it was illegal to kill them with cyanide gas or powder, which was standard practice with burrowing pests such as rabbits and moles. PICL officers instead recommended capturing ‘rogue badgers’ with snare traps, or, if all else failed, using expert marksmen to shoot them.^99^ Occasionally a whole group caused problems, for example when human housing developments brought humans and badgers into uncomfortably close proximity. In such cases the entire sett could be excavated and destroyed. However, this was often ineffective, as badgers persisted in returning to a cleared area.^100^ PICL scientists therefore recommended first trying to drive the animals away using strong-smelling materials such as creosote.^101^

The scientific uncertainty surrounding the badger's role as transmitter of bTB to cattle, combined with a keen awareness of the political implications of the news, led MAFF to commission further scientific investigations. These involved not only MAFF's veterinary and pest-control experts, but also ‘wildlife interests’ external to MAFF: scientists, amateur natural-history groups and badger protection campaigners. They were invited to head off public controversy, an approach which initially had some success.^102^ These new interlocutors awarded very different roles to badgers than did MAFF officials – as a species to be conserved, as repositories of rural values, and as victims of human cruelty in need of protection. Drawing upon their expertise, PCD and veterinary officers began to survey the immediate area in which the sick badgers had been found. Constructing badgers as both disease victims and transmitters, they sought evidence of their infection with bTB through sampling and testing their faeces, and collecting and examining their dead bodies (which were increasingly presented by members of the public).^103^ Maps and data sheets recorded the locations of diseased and healthy badgers, badger setts and cattle herds with varying incidences of TB. They also incorporated features of the terrain and notes on the presence of other wild animals, which were also investigated as potential bTB transmitters.^104^

These field investigations were far from straightforward. Badgers were a poorly understood, nocturnal species that lived underground in inaccessible rural areas. Investigators therefore ‘had the arduous and painstaking task of finding as many setts as possible’ in a 150-square-kilometre area of Gloucestershire ‘which is well populated by badgers and where steep wooded hillsides make searching quite tiring’.^105^ Nevertheless, researchers continued to follow traces of badger bodies, bodily fluids, tracks and behaviour, and to document these traces using maps, photographs, post-mortem and microbiological reports, and numerical data. In so doing, their understanding of the problem changed: not only were other wildlife samples testing negative for TB, but increasing numbers of TB-positive badgers were identified. Officials had initially regarded the occurrence of bTB in badgers as a relatively isolated incident amenable to sett-by-sett solutions, but as the traces mounted, they identified a rapidly escalating disease outbreak affecting multiple parts of the country.^106^

These shifting understandings brought the problem of badger control to the fore and created another new role for the animals, as subjects of government legislation. In 1973, the government passed the Badgers Act. On the one hand the Act responded to the lobbying of naturalists and badger advocates granting the animals specific protections against killing or cruelty. At the same time, it introduced a framework for licensing ‘to kill or take’ the animals for research or conservation purposes, as well as ‘for purposes of preventing the spread of disease’.^107^ While farming and wildlife interests publicly welcomed the Act, it proved unable to achieve these very different purposes at the same time, as shown when badger campaigner Ruth Murray attempted to use it to prosecute a PCD officer and the minister of agriculture on grounds of animal cruelty after a MAFF demonstration of methods for killing badgers.^108^ This incident precipitated further legislative changes that strengthened the licensing framework and made it legal for MAFF officers to kill badgers with cyanide powder blown into setts, which was a standard pest-control method at the time. Early acknowledgements of the circumstantial nature of the evidence linking bTB transmission to badgers faded into the background, to be replaced by reports discussing the ‘important role’ played by badgers ‘in perpetuating the disease’, and public plans for ‘action to eliminate this reservoir of infection’.^109^ By late 1975 a formal badger-culling policy was in place, and the former, rather ad hoc collaborative investigations had been transformed into a formal MAFF research programme into ‘TB in cattle and badgers’, which continued until 1997.^110^ All of these activities generated additional badger traces and made them visible to new sets of human actors, including veterinarians, policymakers, politicians and journalists, while generating further concern among farmers, scientists and campaigners.

Although legislative changes and the commencement of culling operations in 1975 reinforced the badger's role as a threatening vector of disease, MAFF's expanded research effort tacitly acknowledged that its role in propagating bTB was not yet settled. In the years that followed, the circumstantial nature of the evidence was one important source of scientific and policy controversy, but the multiple and contradictory roles assigned to badgers in the early 1970s and publicized via policy documents and in national and local media also drove ethical, political and emotional responses, which fuelled debates over tuberculous badgers that are still ongoing today.

## Conclusion

This article opened by arguing for the need to develop more animal-centred histories of medicine. It highlighted two potential gains from this approach. First, it would enable historians to advance understandings of animals within medicine. As subjects and objects of investigation, the histories of animals would be illuminated through accounts of their lived experiences and analyses of the multiple roles they played within disease investigations, and by conceptualizing them as active historical agents who shaped – and were shaped by – medical ideas, practices, policies, institutions and careers. Second, it could potentially inspire fresh perspectives on what constituted medicine. Through challenging the prevailing assumption that animals were important to medicine only insofar as they influenced human health, this approach would encourage historians to push beyond the much-studied fields of experimental medicine and public health, and explore wider contexts in which animals and medicine were co-constructed. These include the borderlands of medicine, veterinary medicine and the life sciences, where the connections forged by animals challenge the prevailing historical tendency to regard these as discrete and distinctive disciplines (at least as far as their treatment of animals is concerned).

We illustrated these claims using four short vignettes of diseased animals that were subjected to investigation within the domains of zoology/pathology, parasitology/epidemiology, ethology/psychiatry, and wildlife/veterinary medicine. Following Etienne Benson, we reconstructed their histories using the traces they left on the medical-historical record. These traces ranged from museum specimens of monkey bones to photographs of opossum brains, micrographs of *Echinococcus* cysts, maps of the locations of tuberculous badgers, and the ideas, practices and policies that derived from this material. We explained the reasons behind the creation of these traces, the people and methods involved, the interpretations placed on them and the responses that they elicited. This enabled us to illuminate the short and painful lives of nineteenth-century captive lions and monkeys, the disorientation experienced by marsupials in the psychiatric hospital, the habits of wild badgers, and movements of *E. granulosus* within and between human and animal bodies.

Analysing animal traces revealed how medicine shaped animals – by awarding them roles as patients, parasites, pests, disease victims, vectors, experimental subjects or points of comparison with other species. Rickety lion cubs were transformed into patients analogous to rickety children; marsupials were compared to psychiatric patients; tapeworms were tracked through bodies and epidemiological networks and badgers through landscapes. Their investigation involved the application of diverse scientific methods. While experimentation was important, so, too, were methods derived from clinical medicine, hospital medicine, epidemiology, natural history, ethology and ecology. Animal symptoms and behaviours were observed in life and their pathological anatomy scrutinized after death, with the findings compared to those of other diseased species, particularly humans. Animal relationships with humans, other animals and environments were also analysed. Lions were subjected to curative interventions while badgers were targeted for elimination.

In turn, animal bodies and behaviours shaped medicine. Rickety lions and monkeys informed understandings of the pathology and epidemiology of rickets and its management in people, disconcerted marsupials influenced ideas of human psychiatric disease, *E. granulosus* drew attention to culture as an epidemiological factor, and tuberculous badgers provided new explanations for and responses to tuberculosis in cows. Diseased animals influenced the profiles and careers of their investigators, enabling John Bland Sutton to become a leading London surgeon, and Calvin Schwabe to be appointed professor of veterinary epidemiology at the University of California, Davis. They also had institutional impacts: London Zoo became a site of systematic medical research, marsupials took up residence in the Waldau institute of brain anatomy (which was expanded as a consequence), the AUB devoted laboratory space to nurturing *E. granulosus*, while the British Ministry of Agriculture dedicated veterinary, scientific and financial resources to the study of badgers.

Collectively, these vignettes draw attention to people, places, species and scientific methods that rarely feature within existing medical historiography. Few scholars have heard of John Bland Sutton, Giorgio Pilleri or Calvin Schwabe. When they conduct research into the histories of animals in medicine, they typically examine laboratory settings, not London Zoo, the Waldau psychiatric hospital, the human–animal communities of 1950s Beirut, or the landscapes of 1970s Gloucestershire. They focus selectively on the rodents, dogs, monkeys and farmed livestock that were manipulated experimentally and/or implicated in spreading disease to humans, not on the lions, marsupials, tapeworms and mustelids that feature in this article. Likewise, in focusing narrowly upon animals as disease transmitters or experimental material, they fail to recognize the many other roles that animals played within medicine, and the diverse methods that were applied to their investigation within the contexts of zoology/pathology, parasitology/epidemiology, ethology/psychiatry, and wildlife/veterinary medicine.

In starting to uncover these neglected histories, the four vignettes presented here open up new perspectives on medicine – its content and concerns, methods and personnel, institutions and subjects of enquiry, relationships with veterinary medicine and the life sciences, and on the types of animal that helped to forge its history. They reveal that in spite of its dominant position within the medical historiography, experimentalism was just one of many approaches to studying animals. While its default position may prove resistant to challenge (as illustrated by the fact that reviewers of this and similar articles have consistently asked why we do not pay more attention to the history of animal experiments), the evidence presented here illustrates the need and potential for historians to expand their horizons. By situating animals at the heart of their analyses, and following diverse animal species through the diverse domains of medicine, they will develop richer accounts of the past that simultaneously reshape historical understandings of animals, medicine and the relationships between them.

## Footnotes

1 Anne Hardy, ‘Animals, disease and man: making connections’, Perspectives in Biology and Medicine (2003) 46, pp. 200–2151272152110.1353/pbm.2003.0021; Keir Waddington, The Bovine Scourge: Meat, Tuberculosis and Public Health, 1850–1914, Woodbridge: Boydell Press, 2006; Mark Worboys and Neil Pemberton, Mad Dogs and Englishmen: Rabies in Britain, 1830–2000, Basingstoke: Palgrave Macmillan, 2007; Kirk Arden Hoppe, Lords of the Fly: Sleeping Sickness Control in British East Africa 1900–1960, Westport, CT: Praeger, 2003; Susan Jones, Death in a Small Package: A Short History of Anthrax, Baltimore: Johns Hopkins University Press, 2010; Ki-heung Kim, The Social Construction of Disease: From Scrapie to Prion, London: Routledge, 2007.

2 Lise Wilkinson, Animals and Disease: An Introduction to the History of Comparative Medicine, Cambridge: Cambridge University Press, 1992; Anita Guerrini, Experimenting with Humans and Animals: From Galen to Animal Rights, Baltimore: Johns Hopkins University Press, 2003; W. Bynum, ‘“C'est un malade”: animal models and concepts of human diseases’, Journal of the History of Medicine and Allied Sciences (1990) 45, pp. 397–413212032710.1093/jhmas/45.3.397.

3 Jonathan Simon, ‘Monitoring the stable at the Pasteur Institute’, Science in Context (2008) 21, pp. 181–2001883113610.1017/s0269889708001683; Thomas Schlich, Eric Mykhalovskiy and Melanie Rock, ‘Animals in surgery – surgery in animals: nature and culture in animal–human relationship and modern surgery’, History and Philosophy of the Life Sciences (2009) 31, pp. 321–35420586136.

4 Nicolaas Rupke (ed.), Vivisection in Historical Perspective, London: Routledge, 1990; Hilda Kean, ‘The “smooth cool men of science”: the feminist and socialist response to vivisection’, History Workshop Journal (1995) 40, pp. 16–3810.1093/hwj/40.1.1611608961.

5 Adele Clarke and Joan Fujimura (eds.), The Right Tools for the Job: At Work in Twentieth-Century Life Sciences, Princeton, NJ: Princeton University Press, 1992; Bonnie Clause, ‘The Wistar rat as a right choice: establishing mammalian standards and the ideal of a standardized mammal’, Journal of the History of Biology (1993) 26, pp. 329–3491162316410.1007/BF01061973; Karen Rader, ‘Scientific animals’, in Randy Malamamud (ed.), A Cultural History of Animals in the Modern Age, London: Berg, 2007, pp. 119–137.

6 Dan Vandersommers, ‘The “animal turn” in history’, *American Historical Association Blog*, 3 November 2016, at http://blog.historians.org/2016/11/animal-turn-history.

7 Most recently see Robert G.W. Kirk and Michael Worboys, ‘Medicine and species: one medicine, one history?’, in Mark Jackson (ed.), The Oxford Handbook of the History of Medicine, Oxford: Oxford University Press, 2011, pp. 561–577; and Michael Bresalier, Angela Cassidy and Abigail Woods, ‘One health in history’, in J. Zinsstag, E. Schelling, D. Waltner-Toews, M. Whittaker and M. Tanner (eds.), One Health: The Theory and Practice of Integrated Health Approaches, Wallingford: CAB International, 2015, pp. 1–15.

8 Key exceptions are: Daniel Todes, ‘Pavlov's physiology factory’, Isis (1997) 88, pp. 205–4610.1086/3836909325628; Robert Kirk, ‘The invention of the “stressed animal” and the development of a science of animal welfare, 1947–86’, in David Cantor and Edmund Ramsden (eds.), Stress, Shock, and Adaptation in the Twentieth Century, Rochester, NY: University of Rochester Press, 2014, pp. 241–26324600751.

9 Key exceptions are Chris Degeling, ‘Negotiating value: comparing human and animal fracture care in industrial societies’, Science, Technology and Human Values (2009) 34, pp. 77–101; Thomas Schlich and Martina Schlünder, ‘The emergence of “implant-pets” and “bone-sheep”: animals as new biomedical objects in orthopedic surgery (1960s–2010)’, History and Philosophy of the Life Sciences (2009) 31, pp. 433–46620586140; Christoph Gradmann, ‘Robert Koch and the invention of the carrier state: tropical medicine, veterinary infections and epidemiology around 1900’, Studies in History and Philosophy of Biological and Biomedical Sciences (2010) 41, pp. 232–2402093464410.1016/j.shpsc.2010.04.012.

10 Roy Porter, ‘Man, animals and medicine at the time of the founding of the Royal Veterinary College’, in A.R. Mitchell (ed.), History of the Healing Professions, 4 vols., Wallingford: CAB International, 1993, vol. 3, pp. 19–30, 19.

11 Abigail Woods, ‘From one medicine to two: the evolving relationship between human and veterinary medicine in England, 1791–1835’, Bulletin of the History of Medicine (2017), forthcoming10.1353/bhm.2017.0058PMC578832629081431.

12 Michel Callon and J. Law, ‘Some elements of a sociology of translation: domestication of the scallops and the fishermen of St Brieuc Bay’, in J. Law (ed.), Power, Action and Belief: A New Sociology of Knowledge, London: Routledge, 1986, pp. 196–223; Bruno Latour, The Pasteurization of France, Cambridge, MA: Harvard University Press, 1988; E. Fudge, ‘A left-handed blow: writing the history of animals’, in N. Rothfels (ed.), Representing Animals, Bloomington: Indiana University Press, 2002, pp. 3–18; Donna Haraway, Where Species Meet, Minneapolis: University of Minnesota Press, 2007; Dorothee Brantz (ed.), Beastly Natures: Animals, Humans, and the Study of History, Charlottesville: University of Virginia Press, 2010; Hilda Kean, ‘Challenges for historians writing animal–human history: what is really enough?’, Anthrozoos (2012) 25, supplement, pp. S57–S72; various, *Does history need animals?*, *History and Theory* special issue (2013) 52; Susan Nance (ed.), The Historical Animal, New York: Syracuse University Press, 2015; Vinciane Despret, What Would Animals Say if We Asked the Right Questions?, Minneapolis: University of Minnesota Press, 2016.

13 ‘One medicine? Investigating human and animal disease’, Wellcome Trust Programme Grant 092719/Z/10/A, PI Abigail Woods.

14 Kean, op. cit. (12), pp. 61–62.

15 Fudge, op. cit. (12), p. 6.

16 E. Benson, ‘Animal writes: historiography, disciplinarity, and the animal trace’, in L. Kalof and G.M. Montgomery (eds.), Making Animal Meaning, East Lansing: Michigan State University Press, 2011, pp. 3–16, 11.

17 Roger Cooter, ‘The turn of the body: history and the politics of the corporeal’, ARBOR Ciencia, Pensamiento y Cultura (2010) 186(743), pp. 393–405; Ivan Crozier, ‘Bodies in history: the task of the historian’, in Crozier (ed.), The Cultural History of the Human Body, *vol. 6:* 1920–Present, London: Bloomsbury, 2014, pp. 1–20; Pascal Eitler, ‘Animal history as body history: four suggestions from a genealogical perspective’, Body Politics (2014) 2, pp. 259–274.

18 Case 14, Bay 8, Hunterian Museum of the Royal College of Surgeons, London [RCS].

19 RCSPC/01291 – skeleton, rickets, mounted dry bone. Museum Collections, RCS.

20 John Bland Sutton, ‘On the diseases of monkeys in the society's gardens’, Proceedings of the Zoological Society London (1883), pp. 581–586.

21 Such matters rarely feature in histories of this institution. See e.g. Takashi Ito, London Zoo and the Victorians, 1828–1859, Rochester, NY: Boydell and Brewer, 2014.

22 John Bland Sutton, The Story of a Surgeon, London: Methuen, 1931.

23 See, for example, John Bland Sutton, ‘On the diseases of the carnivorous mammals in the society's gardens’, Proceedings of the Zoological Society London (1884), pp. 177–187; Bland Sutton, ‘Comparative dental pathology’, Odontological Society Transactions (1884) 16, pp. 88–145.

24 Bland Sutton, op. cit. (22).

25 H.R. Dean, ‘The Pathological Society of London’, Proceedings of the Royal Society of Medicine (1946) 39(12), pp. 823–8271999341510.1177/003591574603901218PMC2182434.

26 ‘Pathological Society of London’, British Medical Journal (1879) 25(1), pp. 123–125; Pathological Society of London, Minute Book 3, AGM 7/1/79, Royal Society of Medicine Archive, London; W.H. Power, ‘Thoughts on the nature of certain observed relations between diphtheria and milk’, Transactions of the Pathological Society London (1879) 30, pp. 546–551.

27 Michael Worboys, Spreading Germs, Cambridge: Cambridge University Press, 2000.

28 Archives of the Pathological Society of London, Royal Society of Medicine: Council and General Minute Book, 1852–1884, PSL/A/6; Annual Report Book, 1879–1886, PSL/A/R/2.

29 Wilkinson, op. cit. (2).

30 Bland Sutton, op. cit. (20), p. 586.

31 John Bland Sutton, ‘Rickets in a baboon’, Transactions of the Pathological Society of London (1883) 34, pp. 312–315; and Bland Sutton, ‘Bone disease in animals’, ibid., pp. 315–322; ‘Pathological Society of London’, The Lancet (1883) 122(3138), pp. 685–686.

32 J. Bland Sutton, ‘Observations on rickets, &c., in wild animals’, Journal of Anatomy and Physiology (1884) 18(4), pp. 362–387PMC128846217231539.

33 Bland Sutton, op. cit. (32), p. 364.

34 Bland Sutton, op. cit. (32), pp. 381–382.

35 Dr Cheadle, ‘Introductory address: a discussion on rickets’, British Medical Journal (1882) 2(1456), pp. 1145–1148.

36 Bland Sutton, ‘Comparative dental pathology’, op. cit. (23), p. 143.

37 E.g. RCSOM/G 46.12 – skull, rickets, osteomalacia, Museum Collections, RCS.

38 Cheadle, op. cit. (35), p. 1146.

39 Russell W. Chesney and Gail Hedberg, ‘Metabolic bone disease in lion cubs at the London Zoo in 1889: the original animal model of rickets’, Journal of Biomedical Science (2010) 17, Supplement 1, pp. S36–S392080461210.1186/1423-0127-17-S1-S36PMC2994403.

40 G. Pilleri, ‘Bewegungsstereotypien beim nordamerikanischen Opossum’, Revue Suisse de zoologie (1960) 67, pp. 519–521, citing papers by the Swiss zoologists Heini Hediger and Monika Holzapfel and the German psychiatrist Ernst Kretschmer, published in the 1930s.

41 See, for example, Rene Spitz, Harry Harlow, John Bowlby.

42 G. Pilleri, ‘Zum Verhalten der Paka (*Cuniculus paca* Linnaeus)’, Zeitschrift für Säugetierkunde (1960) 25, pp. 107–111.

43 Pilleri, op. cit. (42), p. 109.

44 Bruno Strasser, ‘Collecting nature: practices, styles, and narratives’, Osiris (2012) 27(1), pp. 303–340.

45 Pilleri, op. cit. (40), p. 520.

46 G. Pilleri, ‘Zum Verhalten der *Aplodontia rufa* (Rodentia, Aplodontoidea) in Gefangenschaft’, Zeitschrift für Säugetierkunde (1960) 25(1), pp. 30–345893867; Irene Wandeler and G. Pilleri, ‘Weitere Beobachtungen zum Verhalten von *Aplodontia rufa* Rafinesque (Rodentia, Aplodonoidea) in Gefangenschaft’, Zeitschrift für Tierpsychologie (1965) 22(5), pp. 570–5835893867.

47 Pilleri, op. cit. (40), p. 110.

48 G. Pilleri to Regierungsrat Blaser, 5 August 1965, Staatsarchiv Bern, B Verwaltungsarchive, BB X Bauwesen, BB X 1220: ‘Hirnanatomisches Institut Waldau (1964–1965)’.

49 Ernst Grünthal to W.G. Stoll (Geigy), 16 May 1963, folder ‘Stiftungen’, box ‘Schreibtisch Schubladen Mitte’, Grünthal papers, Schweizerisches Psychiatrie-Museum Bern (Waldau), Bern.

50 G. Pilleri, Das hirnanatomische Institut der psychiatrischen Universitätsklinik Bern: 1934–1969 Rückschau und Ausblick, Bern: Hirnanatomisches Institut, 1969.

51 Lucie Gerber, ‘Marketing loops: the development of psychopharmacological screening at Geigy in the 1960s and 1970s’, in Jean-Paul Gaudillière and Ulrike Thoms (eds.), The Development of Scientific Marketing in the Twentieth Century: Research for Sales in the Pharmaceutical Industry, London: Pickering & Chatto, 2015, pp. 191–212.

52 G. Pilleri, ‘Zur feineren Struktur und vergleichenden Anatomie des Corpus Striatum primitive Beutel- und Nagetiere’, Acta Anatomica (1962), 48, pp. 347–36713943611.

53 See, on a similarly fascinating mammal in American comparative psychology, Michael Pettit, ‘The problem of raccoon intelligence in behaviourist America’, BJHS (2010) 43(3), pp. 391–421.

54 G. Pilleri and K. Poeck, ‘Arterhaltende und soziale Instinktbewegungen als neurologische Symptome beim Menschen’, Psychiatria et Neurologia (1964) 147(4), pp. 193–23814154077.

55 G. Pilleri, ‘Instinktbewegungen des Menschen in biologischer und neuropathologischer Sicht’, Bibliotheca Psychiatrica (1971) 147, pp. 1–374995197, 6.

56 Pilleri, op. cit. (55), p. 2. An argument also made by Konrad Lorenz, whom Pilleri cited. Lorenz had notably made his comments during National Socialism, describing (human) domestication as one of these ultimately limiting forms of overspecialization.

57 Pilleri and Poeck, op. cit. (54), p. 195.

58 Pilleri and Poeck, op. cit. (54), thus cited Constantin von Monakow's theory of chronogenic localization of functions and John Hughlings Jackson's work.

59 Summarized and cited in Pilleri and Poeck, op. cit. (54).

60 In neighbouring France, Henri Ey, ‘Le concept de “psychiatrie animale” (difficultés et intérêt de sa problématique)’, in Abel Brion and Henri Ey (eds.), Psychiatrie animale, Paris: Desclée de Brouwer, 1964, pp. 11–40; Marga Vicedo, ‘The father of ethology and the foster mother of ducks: Konrad Lorenz as expert on motherhood’, Isis (2009) 100(2), pp. 263–2911965349010.1086/599553. Chloe Silverman, ‘“Birdwatching and baby-watching”: Niko and Elisabeth Tinbergen's ethological approach to autism’, History of Psychiatry (2010) 21(2), pp. 176–1892187737110.1177/0957154x10367875.

61 Detlev Ploog, ‘Verhaltensforschung und Psychiatrie’, in H.W. Gruhle, R. Jung, W. Mayer-Gross and M. Müller (eds.), Psychiatrie der Gegenwart: Forschung und Praxis: Band I/1B: Grundlagenforschung zur Psychiatrie: Teil B, Berlin: Springer, 1964, pp. 291–443, 422.

62 Ploog, op. cit. (61), p. 395. See also Detlev Ploog, ‘Experimentelle Verhaltensforschung’, Der Nervenarzt (1966) 37(10), pp. 443–4474963742. See also contemporaneous debates in psychosomatic medicine, which entwined psyche and soma, individual and milieu.

63 H. Hedinger, ‘Über Bewegungs-Stereotypien bei gehaltenen Tieren’, Revue Suisse de zoologie (1934) 41(17), pp. 349–356, 355 f.

64 Among others see E. Frauchiger, Seelische Erkrankungen bei Mensch und Tier: Eine Grundlage für eine vergleichende Psychopathologie, Bern: Hans Huber, 1945; Brion and Ey, op. cit. (60); and contributions in M.W. Fox (ed.), *Abnormal Behaviour in Animals*, Philadelphia: W.B. Saunders, 1968. Jérôme Michalon, ‘Soigner par le contact animalier: aux origines de la recherche sur les interactions humains/animaux à but thérapeutique’, Revue d'histoire des sciences humaines: Les sciences du psychisme et l'animal (2016) 28, pp. 137–162.

65 Richard W. Burkhardt, Patterns of Behavior: Konrad Lorenz, Niko Tinbergen, and the Founding of Ethology, Chicago: The University of Chicago Press, 2005; Erika Loraine Milam, Looking for a Few Good Males: Female Choice in Evolutionary Biology, Baltimore: Johns Hopkins University Press, 2010; Edmund Ramsden and Duncan Wilson, ‘The suicidal animal: science and the nature of self-destruction’, Past & Present (2014) 224(1), pp. 201–2422581477510.1093/pastj/gtu015PMC4367443.

66 Laurel Braitman, Animal Madness: How Anxious Dogs, Compulsive Parrots, and Elephants in Recovery Help Us Understand Ourselves, New York: Simon & Schuster, 2014; Barbara Natterson-Horowitz and Kathryn Bowers, Zoobiquity: What Animals Teach Us about Health and the Science of Healing, New York: Alfred A. Knopf, 2012.

67 F.P. Araujo, C.W. Schwabe, J.C. Sawyer and W.G. Davis, ‘Hydatid disease transmission in California: a study of the Basque connection’, American Journal of Epidemiology (1975) 102(4), pp. 291–302118025310.1093/oxfordjournals.aje.a112164, 298; John C. Sawyer, Peter M. Schantz, Calvin W. Schwabe and Milton W. Newbold, ‘Identification of transmission foci of hydatid disease in California’, Public Health Reports (1969) 84(6), pp. 531–5414977534PMC2031587.

68 Araujo *et al.*, op. cit. (67).

69 Calvin W. Schwabe (*c.*1992), ‘Hoofprints of Cheiron: pursuing a gathered life; Book One: Bugs, worms and all that’, manuscript, vol. 12, p. 359, US National Library of Medicine, Calvin Walter Schwabe Papers, MS C 490.

70 Charles L. Cobgill, ‘*Echinococcus* cyst’, A.M.A. Archives of Surgery (1957) 75(2), pp. 267–2711344363610.1001/archsurg.1957.01280140105019; M.M. Sterman and H.W. Brown, ‘*Echinococcus* in man and dog in the same household in New York City’, JAMA (1959) 169(9), pp. 938–94010.1001/jama.1959.0300026003600813630709; Thomas J. Brooks, Watts R. Webb and Kenneth M. Heard, ‘Hydatid disease: a summary of human cases in Mississippi’, A.M.A. Archives of Internal Medicine (1959) 104, pp. 562–56713804797.

71 Schwabe, op. cit. (69).

72 Carolus Linnaeus, Systema naturae per regna tria naturae: secundum classes, ordines, genera, species, cum characteribus, differentiis, synonymis, locis, 10th edn, Stockholm, Laurentius Salvius, 1758.

73 See, for example, discussion of specialization and adaptation in parasites in W.P. Rogers, The Nature of Parasitism: The Relationship of Some Metazoan Parasites to their Hosts, London: Academic Press, 1962, esp. Chapter 10, ‘The specificity of parasites’; Geoffrey Lapage, Animals Parasitic in Man, rev. edn, New York: Dover Publications, 1963, esp. Chapter 11, ‘Gain and loss for the parasite’; and V.A. Dogiel, General Parasitology, rev. enlarged edn, Edinburgh: Oliver and Boyd, 1964, esp. Chapter 22, ‘Host specificity and the problem of species in parasites’.

74 Parasitology in the twentieth century emerged from late nineteenth-century tropical medicine and helminthology, where it was as much rooted in the practices of zoology and natural history as in the medical goal of disease prevention. John Farley, ‘Parasites and the germ theory of disease’, in Charles Rosenberg and Janet Lynne Golden (eds.), Framing Disease: Studies in Cultural History, New Brunswick, NJ: Rutgers University Press, 1992, pp. 33–49.

75 Calvin W. Schwabe, Lewis A. Schinazi and Araxie Kilejian, ‘Host–parasite relationships in *Echinococcus*: II. Age resistance to secondary *Echinococcus* in the white mouse’, American Journal of Tropical Medicine and Hygiene (1959) 8, pp. 29–3613617592.

76 Ishak Farhan, Calvin W. Schwabe and C. Richard Zobel, ‘Host–parasite relationships in *Echinococcus*: III. Relation of environmental oxygen tension to the metabolism of hydatid scolices’, American Journal of Tropical Medicine and Hygiene (1959) 8(4), pp. 473–47813670374.

77 Benson, op. cit. (16), p. 5.

78 Calvin Schwabe, G.W. Luttermoser, M. Koussa and S.R. Ali, ‘Serial passage of fertile hydatid cysts of *Echinococcus granulosus* in absence of the definitive host’, Journal of Parasitology (1964) 50, p. 26014170761.

79 Jamie Lorimer examines coexistence between humans and their internal occupants through contemporary biomedical research on the human microbiome, characterizing the healthy human body as a ‘multispecies achievement’, host to a diverse population of bacteria, helminthic worms and more. Jamie Lorimer, ‘Gut buddies: multispecies studies and the microbiome’, Environmental Humanities (2016) 8(1), pp. 57–76. This notion of deep ecological and evolutionary entanglement between humans and helminths has roots in early twentieth-century parasitology. See, for example, Samuel T. Darling, ‘The distribution of hookworms in the zoological regions’, Science (1921) 53(1371), pp. 323–3241778368910.1126/science.53.1371.323; or T.W.M. Cameron, ‘Helminth parasites’, Proceedings of the Royal Society of Medicine (1929) 22, pp. 827–8291998698410.1177/003591572902200646PMC2102519.

80 Calvin Schwabe, ‘Host–parasite relationships in *Echinococcus*: I. Observations on the permeability of the hydatid cyst wall’, American Journal of Tropical Medicine and Hygiene (1959), 8, pp. 20–2813617591, 21.

81 Calvin W. Schwabe and Kamal Abou Daoud, ‘Epidemiology of *Echinococcus* in the Middle East, I. Human infection in Lebanon, 1949–1959’, American Journal of Tropical Medicine and Hygiene (1961) 9, pp. 377–37910.4269/ajtmh.1961.10.37413749038, 377.

82 Carl W. Miller, Roger Ruppaner and Calvin W. Schwabe, ‘Hydatid disease in California: study of hospital records, 1960–1969’, American Journal of Tropical Medicine and Hygiene (1971) 20, pp. 904–9135167186.

83 Sawyer *et al.*, op. cit. (67).

84 Irwin K.M. Liu, Calvin W. Schwabe, Peter M. Schantz and Malcolm N. Allison, ‘The occurrence of *Echinococcus granulosus* in coyotes (*Canis latrans*) in the Central Valley of California’, Journal of Parasitology (1970) 56, pp. 1135–11375534027.

85 R.H. Muirhead, ‘Bovine tuberculosis in wild badgers in south Gloucestershire’, State Veterinary Journal (1972) 27, pp. 197–205, 198.

86 Peter Atkins, Liquid Materialities: A History of Milk, Science and the Law, London: Ashgate, 2010; Keir Waddington, ‘To stamp out “so terrible a malady”: bovine tuberculosis and tuberculin testing in Britain, 1890–1939’, Medical History (2004) 48, pp. 29–481496864410.1017/s0025727300007043PMC546294.

87 For example, W.D. Macrae, ‘The eradication of bovine tuberculosis in Great Britain’, Tuberculosis in Animals: Symposia of the Zoological Society of London (1960) 4, pp. 81–90.

88 R.A. Richards, W.A Watson, E. Boughton and K. Meldrum, Inquiry into Tuberculosis in West Cornwall, London: Ministry of Agriculture, Fisheries and Food, 1972.

89 Muirhead, op. cit. (85).

90 B. Silcock, ‘Infected badgers face extermination’, *Sunday Times*, 23 April 1972, press clipping in National Archives MAF 145/21, Item 104; F. Butcher, ‘TB time-bomb under a county's dairy farmers’, *Farmer's Weekly*, 22 June 1973, press clipping in National Archives MAF 109/382, Item 89.

91 M.H. Sullivan, 21 February 1972, National Archives, UK (NA), Ministry of Agriculture and Fisheries, Animal Health and Welfare, MAF 287/572, Item 30.

92 J.W. Blackwood, 27 August 1971, NA, Nature Conservancy Council, Conservation, FT 41/88.

93 Angela Cassidy, ‘Vermin, victims and disease: UK framings of badgers in and beyond the bovine TB controversy’, Sociologia Ruralis (2012) 52, pp. 192–214.

94 Patrick Barkham, Badgerlands: The Twilight World of Britain's Most Enigmatic Animal, London: Granta Books, 2014.

95 Dr J.F.D. Frazer, 8 September 1971, NA, FT 41/88; Blackwood, op. cit. (92).

96 J. Steere-Williams, ‘Performing state medicine during its “frustrating” years: epidemiology and bacteriology at the local government board, 1870–1900’, Social History of Medicine (2014) 28, pp. 82–107.

97 Dr P.A. Gay, 23 March 1972, NA, FT 41/88; Registry, Extract from Unconfirmed Minutes of Scientific Policy Committee, Nature Conservancy, 11 July 1972, NA, FT 41/88.

98 See W. Grant, ‘Intractable policy failure: the case of bovine TB and badgers’, British Journal of Politics and International Relations (2009) 11, pp. 557–573 – this ongoing disagreement over whether or not badgers were pests explains why PCD officers appeared defensive of the animals.

99 For example, E.J. Rickot, 5 November 1965, NA, Ministry of Agriculture and Fisheries, Control of Animal and Bird Pests Series, MAF 131/70, Item 112.

100 N.J. Manfield, 24 October 1972, NA, Ministry of Agriculture and Fisheries, Gloucester Divisional Office, MAF 145/21, Item 146; ‘Bread and honey bait traps Cam's badgers’, *Dursley Gazette*, 4 November 1972, press clipping in National Archives MAF 109/382, Item 1.

101 J. Winfield, 14 March 1969, NA, MAF 145/21, Item 74.

102 W.E. Mason, 17 February 1972, NA, MAF 287/572, Item 40.

103 The combination of these logistical/financial pressures and the enthusiasm of publics was such that from 1976 onwards a formal ‘badger survey’ was instituted by MAFF, whereby found badger carcasses were tested for bTB and the data were recorded nationwide until 1997. Today such a project would undoubtedly be described as ‘citizen science’; see, for example, Sally Shuttleworth, ‘Old weather: citizen scientists in the 19th and 21st centuries’, Science Museum Group Journal (2016) 3(3), accessed at doi:10.15180/150304.

104 R.H. Muirhead, J. Gallagher and K.J. Burn, ‘Tuberculosis in wild badgers in Gloucestershire: epidemiology’, Veterinary Record (1974) 95, pp. 552–555446273510.1136/vr.95.24.552.

105 R.J. Clark, 18 July 1972, NA, MAF 145/21, Item 109.

106 A.C.L. Brown, Animal Health 1974: Report of the Chief Veterinary Officer, London: Ministry of Agriculture, Fisheries and Food, 1974, pp. 46–47.

107 *Badgers Act 1973*, London: HM Stationary Office, p. 4.

108 ‘The agony of a badger’, *Daily Mail*, 29 January 1975, p. 10.

109 A.C.L. Brown, Animal Health 1975: Report of the Chief Veterinary Officer, London: Ministry of Agriculture, Fisheries and Food, 1975, pp. 1, 14–15.

110 *Bovine Tuberculosis in Badgers: A Report by the Ministry of Agriculture, Fisheries and Food*, London: Ministry of Agriculture, Fisheries and Food, November 1976.

